# Water transport mediated by murine urea transporters: implications for urine concentration mechanisms

**DOI:** 10.1242/bio.051805

**Published:** 2020-08-14

**Authors:** J. Kabutomori, N. Pina-Lopes, R. Musa-Aziz

**Affiliations:** Department of Physiology and Biophysics, Institute of Biomedical Sciences, University of Sao Paulo, Sao Paulo, Brazil 05508-900

**Keywords:** Urea transporter, Water transport, Concentrated urine, *Lithobates* oocytes, Membrane permeability, Renal physiology

## Abstract

Urea transporters (UTs) facilitate urea diffusion across cell membranes and play an important role in the urinary concentration mechanisms in the kidney. Herein, we injected cRNAs encoding for c-Myc-tagged murine UT-B, UT-A2 or UT-A3 (versus water-injected control) in *Lithobates* oocytes and evaluated oocyte surface protein expression with biotinylation and immunoblotting, urea uptake using [^14^C] counts and water permeability (P*_f_*) by video microscopy. Immunoblots of UT-injected oocyte membranes revealed bands with a molecular weight consistent with that of a UT monomer (34 kDa), and UT-injected oocytes displayed significantly increased and phloretin-sensitive urea uptake and P*_f_* when compared to day-matched control oocytes. Subtracting the water-injected urea uptake or P*_f_* values from those of UT-injected oocytes yielded UT-dependent values*. We demonstrate for the first time that UT-A2 and UT-A3 can transport water, and we confirm that UT-B is permeable to water. Moreover, the [^14^C] urea*/P_f_* ratios fell in the sequence mUT-B>mUT-A2>mUT-A3, indicating that UTs can exhibit selectivity to urea and/or water. It is likely that specific kidney regions with high levels of UTs will exhibit increased urea and/or water permeabilities, directly influencing urine concentration. Furthermore, UT-mediated water transport activity must be considered when developing UT-inhibitors as novel diuretics.

This article has an associated First Person interview with the first author of the paper.

## Abstract (precis)

**Summary:** Renal urea transporters can transport both urea and water, which could have direct effects on renal function, especially the production of concentrated urine.

## INTRODUCTION

The ability of the kidney to excrete urine more concentrated than the extracellular fluid depends on the formation of a hyperosmotic renal medullary interstitium, which provides an osmotic driving force that favors water reabsorption from the collecting duct (CD) system; a process controlled by the presence of antidiuretic hormone (ADH) (Berliner and Bennett, 1967; Giebisch et al., 2017; Knepper, 1997; Knepper et al., 2015). The high osmolarity in the renal medulla is mainly established by the accumulation of NaCl, reabsorbed in the thick ascending limb (TAL) of the Loop of Henle (Ares et al., 2011; Gamba et al., 1994; Payne and Forbush, 1994; Greger and Schlatter, 1981), and urea, reabsorbed in the inner medullary CD (IMCD) (Knepper et al., 2015; Knepper and Roch–Ramel, 1987; Knepper and Star, 1990; Morgan and Berliner, 1968). It is well known that ADH upregulates the functional expression of the water channel aquaporin 2 (AQP2) into the apical membrane of the CD principal cells, thereby greatly increasing the apical membrane’s water permeability (and thus the overall water permeability of the epithelium), playing a critical role in urine concentration (Fushimi et al., 1993; Harris et al., 1991; Knepper et al., 2015; Nielsen et al., 1993). Previous studies have shown that ADH also stimulates the NaCl reabsorption in the TAL via modulation of Na-K-2Cl cotransporter (NKCC2) activity (Molony et al., 1987; Sun et al., 1991) and the urea reabsorption in the IMCD via upregulation of urea-transporters (UTs) expression (Knepper et al., 2015; Sands et al., 2011, 1987; Stewart et al., 2009; Wade et al., 2000; Zhang et al., 2002), increasing the formation of the osmotic gradient necessary for water reabsorption from the CD system (Knepper, 1997; Knepper et al., 2015).

With regard to urea accumulation in the medullary interstitium, the concentration of this compound depends on an urea recycling process, during which urea is freely filtered by the glomerulus, reabsorbed by the proximal tubule (PT), secreted into the thin descending limb (TDL) of the Loop of Henle and reabsorbed by the IMCD (Knepper et al., 2015; Lassiter et al., 1961; Sands et al., 1987; Uchida et al., 2005). Subsequent studies discovered that specific transmembrane UTs facilitate the transport of urea down its concentration gradient across plasma membranes in certain regions of the kidney (Karakashian et al., 1999; Lucien et al., 2005; Shayakul et al., 1996; Stewart et al., 2009; You et al., 1993).

The UTs are members of the SLC14 family of solute carriers. Mammals possess two UT genes, *SLC14A1*, which encodes for the UT-B isoform, and *SLC14A2*, which encodes for the UT-A isoforms (Olives et al., 1994; You et al., 1993). In the kidneys of mice, UT-B has been shown to be localized in the descending vasa recta (DVR) in the inner renal medulla (Lucien et al., 2005; Pallone, 1994; Xu et al., 1997). The renal UT-A isoforms include UT-A1 in the apical membrane of the IMCD (Shayakul et al., 1996), UT-A2 in the TDL of the Loop of Henle (Lei et al., 2011; You et al., 1993) and UT-A3 in the basolateral membrane of the IMCD (Terris et al., 2001). It should be pointed out that UT-A2 and UT-A3 correspond to the C- and N-terminal halves of UT-A1, thus suggesting some level of transcriptional and/or post-translational regulation (Karakashian et al., 1999).

Crystal structures of the bacterial UT-B homolog dvUT (Levin et al., 2009) and bovine UT-B have been solved (Levin et al., 2012). Both structures revealed that this integral membrane protein assembles into a homotrimer, with each monomer forming an independent urea channel. Using site-directed mutagenesis and molecular dynamics simulations, the authors identified a selectivity filter that forms along the urea pore (Levin et al., 2012). The filter can accommodate multiple dehydrated urea molecules in a single file and effectively transports urea across the membrane, while at the same time excluding charged species like protons, ammonium and guanidinium (Levin et al., 2012, 2009).

However, the most compelling evidence for the essential role of UTs in the urine concentration mechanism comes from physiological studies with knockout mice. For example, UT-B knockout mice fed with a normal diet presented increased urinary flow and low urine osmolarity, indicating a defect in the urine concentrating mechanism (Smith, 2009; Yang and Verkman, 2002). Additionally, UT-A2 knockout mice fed with a low protein diet or water restricted exhibit significantly reduced urinary concentration (Smith, 2009; Uchida et al., 2005). Studies with UT-A1 and UT-A3 double knockouts (Fenton et al., 2004; Smith, 2009) and a novel mouse model, in which all UTs were knocked out (Jiang et al., 2017), reported significantly increased water intake and urine flow and reduced urinary osmolarity when compared to wild-type animals. Interestingly, the UTs-null mice were unable to properly regulate urinary urea concentration and osmolarity following water restriction, acute urea loading or high protein intake (Jiang et al., 2017). The authors also reported reduced blood pressure and essentially no physiological abnormalities in the extrarenal organs. Indeed, this specific disorder in the ability of the kidney to concentrate urine in UTs knockout mice supports the rationale behind the development of UT inhibitors as novel diuretics (Esteva-Font et al., 2013; Zhang et al., 2018).

While it is clear that renal UTs mediate the transport of urea essential for the urinary concentrating mechanism, there are some conflicting reports over whether or not these membrane proteins also mediate the transport of water. For example, experiments performed with *Xenopus laevis* oocytes reported that mammalian UT-B not only transports urea but also water (Geyer et al., 2013a; Yang and Verkman, 1998) and ammonia (Geyer et al., 2013a). Notably, urea, water and ammonia transport were all inhibited after treating the oocytes with the well known UT-inhibitor phloretin (Geyer et al., 2013a; Yang and Verkman, 1998), indicating that all three molecules use the same molecular pathway, the urea pore. In contrast, another study using stopped-flow light scattering experiments with enriched *Xenopus* oocyte plasma membrane vesicles containing murine UT-A2 (mUT-A2) or mUT-A3, demonstrated that these vesicles were permeable to urea, but not to water, ammonia or other urea-related molecules (MacIver et al., 2008).

Given the strategic renal localization of UTs and the previously observed water transport function of UT-B, the present study sought to investigate the urea uptake and water permeability of *Lithobates* oocytes (Kabutomori et al., 2018) expressing c-Myc-tagged mUT-B, mUT-A2 and mUT-A3. The results confirm that all three UTs can transport urea, that mUT-B can transport water and also demonstrate for the first time that mUT-A2 and mUT-A3 conduct water. UTs-mediated urea and water transports were significantly inhibited by phloretin. The computed UT-dependent [^14^C] urea*/P_f_* ratio fell in the sequence mUT-B>mUT-A2>mUT-A3. Thus, it is not unreasonable to speculate that the strategic expression of different UT isoforms in specific regions of the kidney could modulate the membrane permeability to urea and/or water for optimal concentrated urine production. Furthermore, the apparently shared molecular pathway urea and water use must be taken into consideration when developing UT-inhibitor-based diuretics that could impair urinary concentrating function.

## RESULTS

### Western blot

**Fig. 1.**
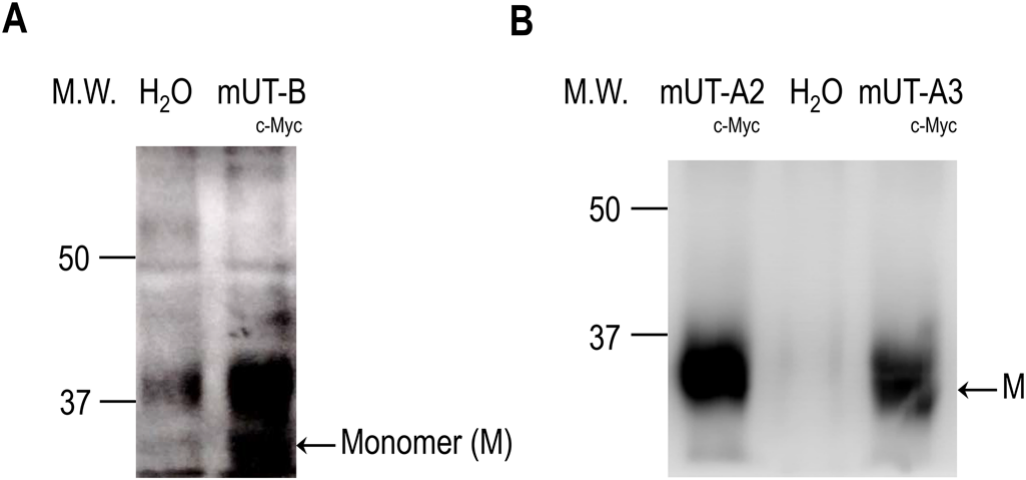
**Membrane expression of mUT-B_,_ mUT-A3 and mUT-A2 in *Lithobates* oocytes.** Immunoblots of biotinylated samples from oocytes injected with cRNA encoding for (A) mUT-B, (B) mUT-A2 and mUT-B, along with H_2_O-injected controls. The representative blots of four independent experiments demonstrate the heterologous expression and insertion into the oocyte membrane. Immunoreactive bands were detected with a monoclonal antibody against the cMyc-tag of the UTs. Each tagged protein was expected to have a MW of approximately 34 kDa and is consistent with the MW of the UT monomers (M) identified in this study. Biotinylated H_2_O-injected controls displayed no immunoreactivity in this region.

Using our previously described surface lysine biotinylation tagging method (Kabutomori et al., 2018), and taking advantage of the C-terminal c-Myc tag, we were able to evaluate the surface expression of mUT-B, mUT-A2 and mUT-A3 in *Lithobates* oocytes (Fig. 1). Immunoreactive bands in the surface biotinylated samples from mUT-B (Fig. 1A, right lane), mUT-A2 (Fig. 1B, left lane) and mUT-A3 (Fig. 1B, right lane) cRNA-injected oocytes occurred at an apparent molecular weight (MW) of 34 kDa, which is consistent with the predicted MW of the c-Myc-tagged UT monomers (Karakashian et al., 1999; Lucien et al., 2005; MacIver et al., 2008). In contrast, surface biotinylated samples from H_2_O-injected control oocytes produced little (Fig. 1A, left lane) or no (Fig. 1B, middle lane) immunoreactivity at this MW.

### Urea uptake

**Fig. 2.**
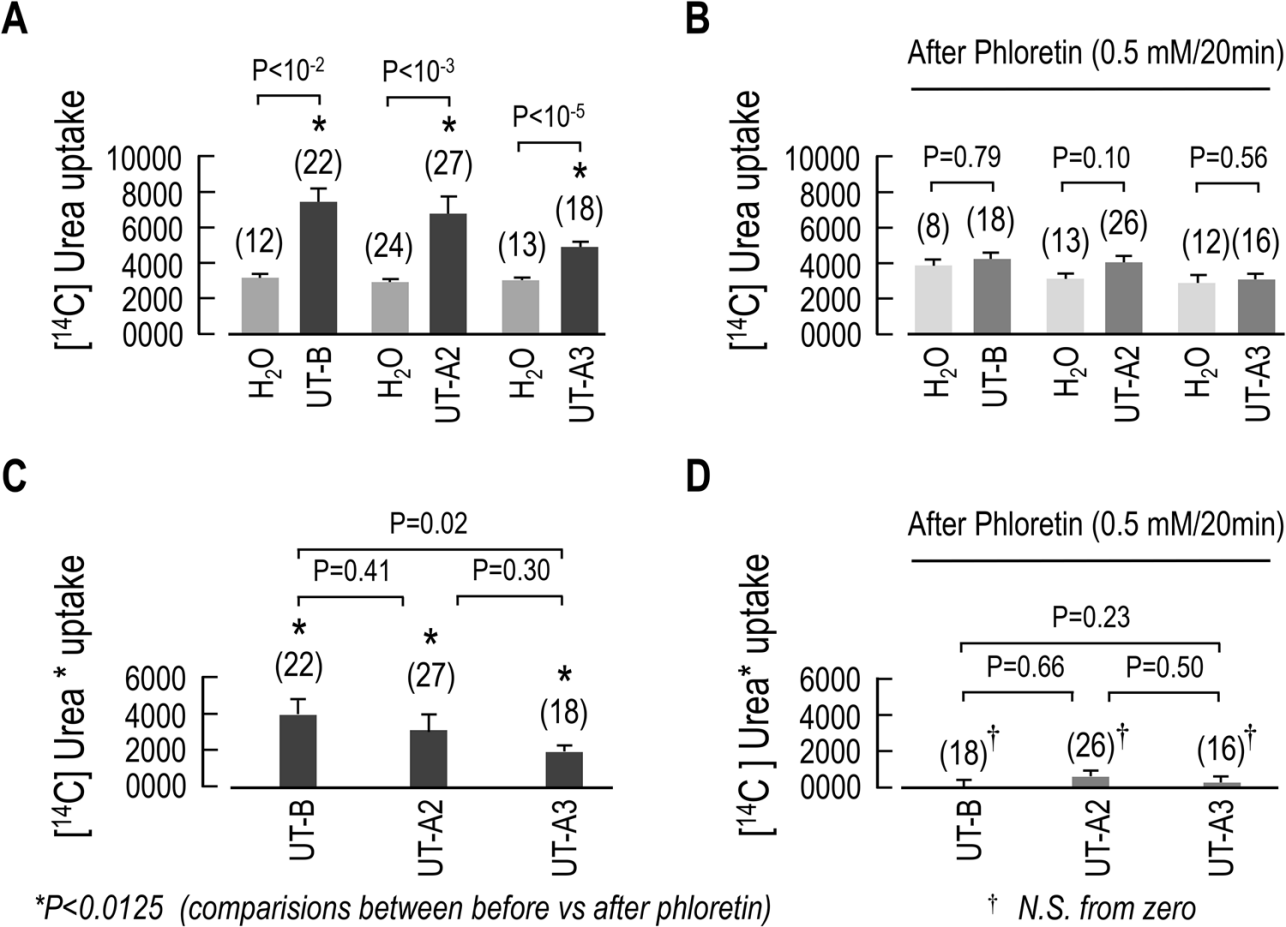
**Summary of the [C^14^] Urea uptake in oocytes expressing mUT-A2_,_ mUT-A3 or mUT-B.** (A) [^14^C] urea uptake measurements with UT-injected oocytes and day-matched H_2_O-injected controls. (B) Effect of phloretin (0.5 mM for 20 min) on [^14^C] urea uptake of UT expressing oocytes and day-matched H_2_O-injected controls. (C) UT-dependent [^14^C] urea uptake ([^14^C] urea*) before and (D) after phloretin treatment. Data are presented as the mean±s.e.m. The number of oocytes (n) used for each set of data is in parentheses above the respective bar graph. It was performed a Student's *t*-tests (*P* shown for individual comparisons). *, comparisons between before and after phloretin treatment (black bars in Fig. 2A and dark grey bars in Fig. 2B) (black bars in Fig. 2C and dark grey bars in Fig. 2D), using Student's *t*-tests with Bonferroni correction (*P*-values of ≤0.0125).

After confirming that the UTs could be transcribed and inserted into the oocyte membrane, we evaluated functional expression by measuring [^14^C] urea uptake of UT- and H_2_O-injected oocytes. We found that oocytes expressing mUT-B, mUT-A2 or mUT-A3 displayed significantly higher [^14^C] urea uptake when compared to the day-matched H_2_O-injected controls (Fig. 2A, comparisons between grey and black bars). To confirm that the enhanced [^14^C] urea uptake was mediated by UTs, we assessed this process following phloretin inhibition (Chou and Knepper, 1989; Esteva-Font et al., 2013; Fenton et al., 2004). It is well known that UT-mediated urea transport is inhibited by phloretin (Geyer et al., 2013a; Yang and Verkman, 1998). As shown in Fig. 2B, UT-injected oocytes pretreated with 0.5 mM phloretin for 20 min (dark grey bars) exhibited reduced [^14^C] urea uptake when compared to similarly treated day-matched H_2_O-injected controls (light grey bars). In fact, [^14^C] urea UT-mediated uptake was attenuated to levels not significantly different from H_2_O control oocytes. Subtracting the [^14^C] urea uptake value for each UT-expressing oocyte from the mean [^14^C] urea uptake of day-matched H_2_O-injected controls yields the UT-dependent [^14^C] urea uptake ([^14^C] urea*). As shown in Fig. 2C, the [^14^C] urea* was augmented in mUT-B, mUT-A2 and mUT-A3 injected oocytes (Fig. 2C). Additionally, phloretin treatment reduced the UT-[^14^C] urea* to a value not different from zero (Fig. 2D).

### Osmotic water permeability

Previously, our *Lithobates* expression system demonstrated that these oocytes were a viable alternative for water-transport studies (Kabutomori et al., 2018), and earlier studies have shown that UT-B expressing *Xenopus* oocytes display increased P*_f_* values (Geyer et al., 2013a; Yang and Verkman, 1998). Therefore, we not only sought to provide additional insights into whether or not UT-B mediates water transport, but to also evaluate the water transport capabilities of UT-A2 and UT-A3. Using video microscopy to monitor the rate of oocyte swelling following exposure to a hypotonic variant of the ND96 solution, we were able to monitor changes in the volume of the oocyte over time and calculate the P*_f_* (cm/s).

**Fig. 3.**
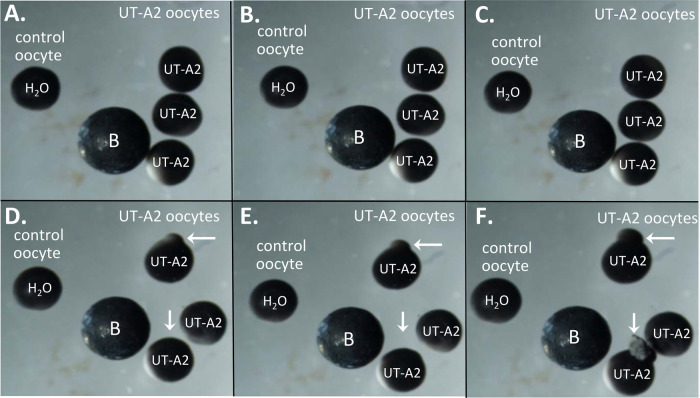
**Representative time course of cellular swelling with mUT-A2-injected and H_2_O-injected control oocytes.** (A–F) In this particular experiment, three mUT-A2-injected oocytes and one H_2_O-injected control were exposed to a hypotonic ND96 variant solution (∼70 mOsm) over the time course of 5 min. (A) Zero time point and (B–F) images collected at minutes 1–5, respectively. A metal ball bearing (labeled B) was included as a measuring reference.

As shown in Fig. 3A–F, representative time-elapsed photos of three mUT-A2-injected oocytes (right side) and one H_2_O-injected control oocyte (left side) exposed to the hypotonic ND96 solution (70 mosmol/l) over the course of 5 min. By the end of the time course, the oocytes expressing UT-A2 swelled and exploded, while no significant changes in oocyte volume were detected in the H_2_O-injected oocyte.

**Fig. 4.**
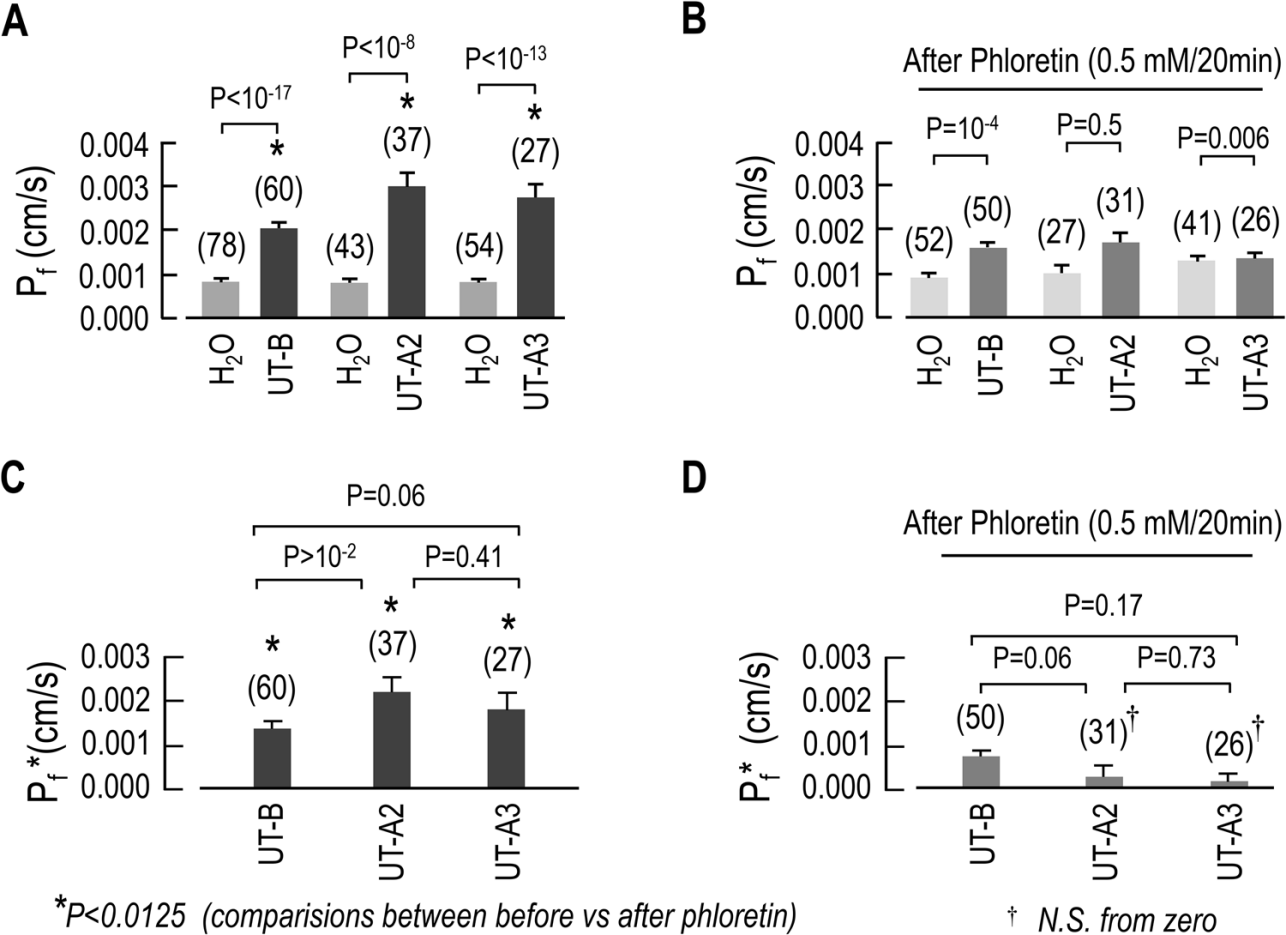
**Summary of the osmotic water permeability (*P*_f_) of oocytes expressing mUT-A2_,_ mUT-A3 or mUT-B.** (A) Osmotic water permeability (*P*_f_) of UTs-injected oocytes and day-matched H_2_O-injected controls. (B) P*_f_* of UT expressing oocytes and day-matched H_2_O-injected controls, following treatment with 0.5 mM phloretin for 20 min. (C) Channel-dependent P*_f_* (P_f_*) before and (D) after phloretin treatment. Data are presented as the mean±s.e.m. The number of oocytes (n) used for each set of data is in parentheses above the respective bar graph. It was performed a Student's *t*-tests (*P* shown for individual comparisons). *, comparisons between before phloretin (black bars in Fig. 4A and dark grey bars in Fig. 4B) and after phloretin treatment (black bars in Fig. 4C and dark grey bars in Fig. 4D), using Student's *t*-tests with Bonferroni correction (*P*-values of ≤0.0125).

Using a larger number of experiments, it was determined that mUT-B, mUT-A2 or mUT-A3-injected oocytes presented significantly higher mean P*_f_* values when compared to the mean of day-matched H_2_O-injected controls (Fig. 4A). To ascertain whether or not these augmented mean P*_f_* values are mediated by heterologous UT expression, the same oocytes used in Fig. 4A were incubated in ND96 containing 0.5 mM phloretin for 20 min, washed and placed in the hypotonic ND96 to a second P*_f_* measurement. Similar to urea transport, phloretin treatment significantly reduced the mean P*_f_* of mUT-B, mUT-A2 and mUT-A3-injected oocytes and had no effect on the day-matched H_2_O-injected controls (Fig. 4B). The UT-dependent P*_f_* (P_f_*) or functional expression was calculated by subtracting from the P*_f_* value of each UT-injected oocyte the mean P*_f_* value of day-matched H_2_O-injected controls. The Pf* results showed that not only did UT expression enhance water transport (Fig. 4C) but also that this activity was phloretin sensitive (Fig. 4D).

**Fig. 5.**
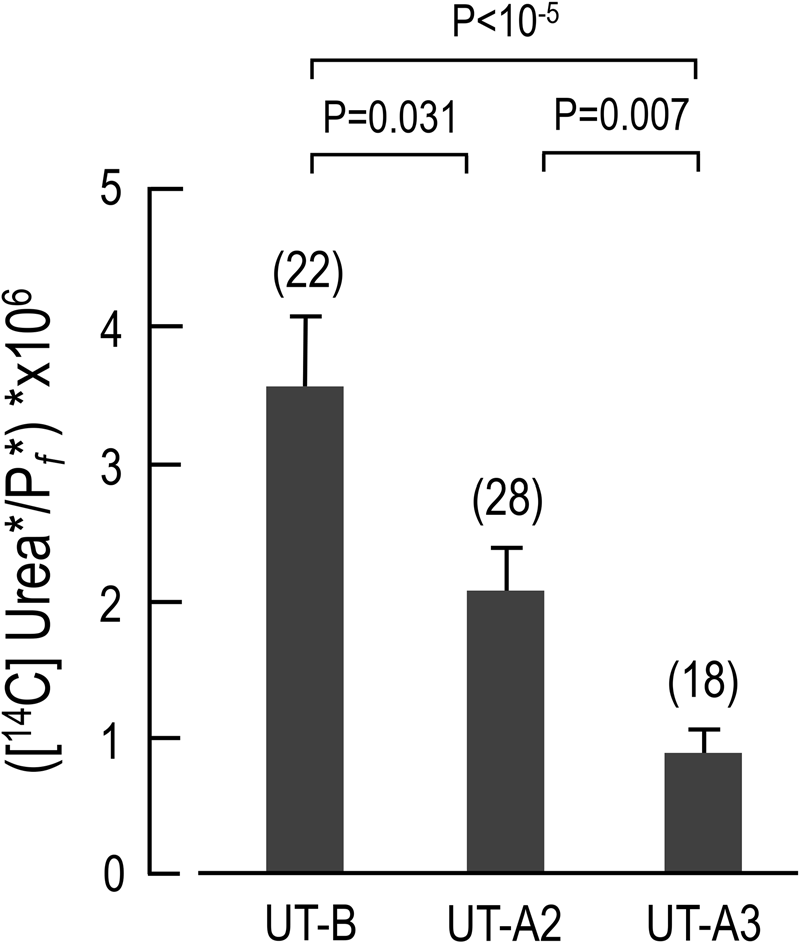
**Urea/water permeability ratios.** For each UT oocyte, the [^14^C] urea* was divided by its P_f_*. Each bar represents the mean±s.e.m. (*n*, number of experiments).

In order to determine whether UTs exhibit selectivity for one substrate over another, which in this case is urea and water, we calculated the urea/water permeability ratio ([^14^C] Urea*/P_f_*) for each UT by dividing the [^14^C] Urea* by P_f_*. The bar graph in Fig. 5 shows that UT-B has the highest [^14^C] Urea*/P_f_* ratio, followed by UT-A2 and UT-A3, indicating that UT-B selectively transports urea (the substance in the numerator) over water (the substance in the denominator), while UT-A2 transports both urea and water similarly, and UT-A3 selectively transports water over urea.

## DISCUSSION

Recently, we demonstrated that *Lithobates catesbeianus* oocytes are a suitable and readily accessible heterologous expression system for evaluating protein-mediated increases in P*_f_* (Kabutomori et al., 2018). In the present study, we evaluated the expression and function of three murine members of the UT family, mUT-B, mUT-A2 and mUT-A3, in these *Lithobates* oocytes. It was determined that all three UTs were expressed at the surface of the oocyte membrane (Fig. 1). It should be pointed out that while it is possible that any protein with a solvent accessible lysine is a potential target for biotinylation and subsequent purification, our protocol employs a highly specific monoclonal antibody developed against the c-Myc tag located on the C-terminal end of each UT (MacIver et al., 2008), which, consequently, enhances protein-detection specificity and reduces the appearance of non-specific bands.

Having established that the *Lithobates* oocytes were heterologously expressing mUT-B, mUT-A2 or mUT-A3 and given that the primary function of UTs is to transport urea, we next evaluated [^14^C] urea uptake with UT-injected oocytes. As expected, mUT-B, mUT-A2 or mUT-A3-injected oocytes displayed significantly higher [^14^C] urea uptake levels that could be inhibited with phloretin. Notably, the [^14^C] urea uptake of both the UT- and H_2_O-injected oocytes was higher than previously reported in *Xenopus* oocytes (Yang and Verkman, 1998; Fenton et al., 2002; Geyer et al., 2013a,). This discrepancy is most likely due to differences in the intrinsic urea permeability of the *Lithobates* oocytes. In addition, the UT-injected oocytes also displayed significantly increased P*_f_* values, which were reduced to just above the control background level by pretreating the oocytes with the UT-inhibitor phloretin. Taken together, the urea uptake and P*_f_* results confirm the functional expression of UT proteins on the surface of the oocyte.

Previously, UT-B-mediated water transport was reported in *Xenopus* oocytes (Geyer et al., 2013a; Yang and Verkman, 1998), and then the Verkman group later reported, using AQP1 and UT-B single and double knockout mice, that UT-B contributes to the movement of water across the erythrocyte membrane (Yang and Verkman, 2002). Notably, Sidoux-Walter et al. (1999) reported that injecting *Xenopus* oocytes with high amounts (40 ng) of human UT-B cRNA increased the both the urea uptake and P*_f_*. However, under these conditions, only the P*_f_* was sensitive to phloretin. On the other hand, when they injected low amounts (0.1 ng) of cRNA, the urea uptake could be inhibited by phloretin and P*_f_* was no longer detectable, thus suggesting that physiological urea transport characteristics are only observed when low amounts of cRNA are injected into the *Xenopus* oocytes. In the present study, we injected 25 ng of cRNA into *Lithobates* oocytes and were not only able to detect UT-B expression, but also significantly increased urea and water transport activities that were both phloretin sensitive. While we are unable to ascertain whether UT-B expression is comparable to the protein expression levels in the murine kidney, our experimental model recapitulates physiological urea transport and provides further evidence in favour of UT-B-mediated water transport.

Additionally, we were able to show, for the first time, that UT-A2 and UT-A3 can also increase the P*_f_* of the oocytes. Considering that phloretin inhibited both urea and water transport through UTs, it is almost certain that both molecules permeate the cell membrane via the same pathway (i.e. the urea pore) and likely rely on similar, or perhaps identical molecular mechanisms. It should be mentioned that another report failed to detect any osmotically-driven changes in the P*_f_* of purified *Xenopus* oocyte plasma membrane vesicles containing UT-A2 and UT-A3 (MacIver et al., 2008). It is plausible that the different experimental procedures (i.e. vesicle preparation versus frog oocytes) and/or approaches (i.e. hypertonic shrinkage versus hypotonic swelling) account for the divergent results.

Furthermore, since UT-A2 and UT-A3 correspond to the C- and N-terminal halves of UT-A1 (Karakashian et al., 1999), it is plausible that UT-A1 is also water permeable. However, we were unable to successfully express UT-A1 in *Lithobates* oocytes, despite devoting a considerable amount of time and resources to this effort. The exact reasons for this lack of expression are unknown, but could be related to the large size of the cRNA transcript and/or translated protein. It should be emphasized that the *Lithobates* expression system is still in its infancy (Kabutomori et al., 2018) and its capabilities and limitations have not been fully elucidated. In addition to NaCl, urea is a major contributor to the high osmolality of the inner medullary interstitium. Indeed, earlier studies have shown that UTs are strategically located in the regions of the kidney that are responsible for creating and maintaining highly concentrated amounts of urea in the medullary interstitium (Karakashian et al., 1999; Lucien et al., 2005; Shayakul et al., 1996; Stewart et al., 2009; You et al., 1993). The increased inner medullary interstitial urea concentration is dependent on a urea-recycling process that is mostly carried out by UT-B, UT-A1, UT-A2 and UT-A3 (Sands and Layton, 2009).

Briefly, the IMCD has relatively high urea permeability due to the presence of the UT-A1 (apical membrane) and UT-A3 (basolateral and apical membrane), promoting urea reabsorption from the IMCD to the medullary interstitium (Hwang et al., 2010). The accumulation of urea in the medullary interstitium drives some of this urea into the medullary TDL through UT-A2 and some to the DVR through UT-B. Once the urea is secreted into the TDL and reaches the IMCD, it can re-enter the medullary interstitium through UT-A1 and UT-A3. Interestingly, Klein et al. (2016) demonstrated that transgenic mice lacking UT-A3 but not UT-A1 exhibit a basal urea permeability that is similar to wild-type mice, which suggests that the presence of UT-A1 is sufficient for maintaining basal levels of urea transport. However, there was a significant reduction in ADH-stimulated urea permeability in the transgenic mice when compared to wild-type mice. Due to the fact that UT-A3 is detected in both the basolateral and apical membranes of the IMCD in the presence of ADH (Hwang et al., 2010; Klein et al., 2016), it appears as though UT-A3 expression in the apical membrane is essential for increasing the urea permeability and P*_f_* of the IMCD in the presence of ADH. Consequently, increasing the interstitial osmolality, which will, in turn, drive water out of the CD system in the presence of ADH, thereby concentrating the urine (Stewart, 2011).

It is known that UTs and AQPs are essential proteins in a variety of physiological processes, including but not limited to the urinary concentrating mechanism to minimise water loss while eliminating waste products (Sands and Layton, 2009). However, it is currently unknown whether or not UT-mediated water transport is physiologically relevant. It has been proposed previously that UT-B, along with AQP1, contributes to water transport from the renal medulla to the vasa recta (Geyer et al., 2013a). Additionally, the TDL limb of the short Loop of Henle is a nephron segment that has been shown to be highly permeable to water under physiological, hydrated or dehydrated conditions, but is essentially devoid of AQP1, and expresses UT-A2 (Zhai et al., 2007). Moreover, under prolonged antidiuretic conditions, UT-A2 has also been shown to be expressed in the base of the inner medullary TDL (Lei et al., 2011). In light of our results, upregulated UT-A2 expression in the TDL could compensate for the lack of AQP-mediated water transport.

Interestingly, when we calculated the [^14^C] Urea*/P_f_* ratios of each UT there were some noticeable differences. For example, UT-B appeared to be more selective towards urea, UT-A2 transported both molecules similarly and UT-A3 favored water. This trend in selectivity is consistent with the localisation of UT-A2 and UT-A3 in nephron segments, the TDL and IMCD, respectively, which are responsible for reabsorbing a large amount of water. In fact, it is plausible that strategically localising different UT isoforms to different regions of the kidney could modulate the production of concentrated urine and consequently regulate the body water balance.

**Fig. 6.**
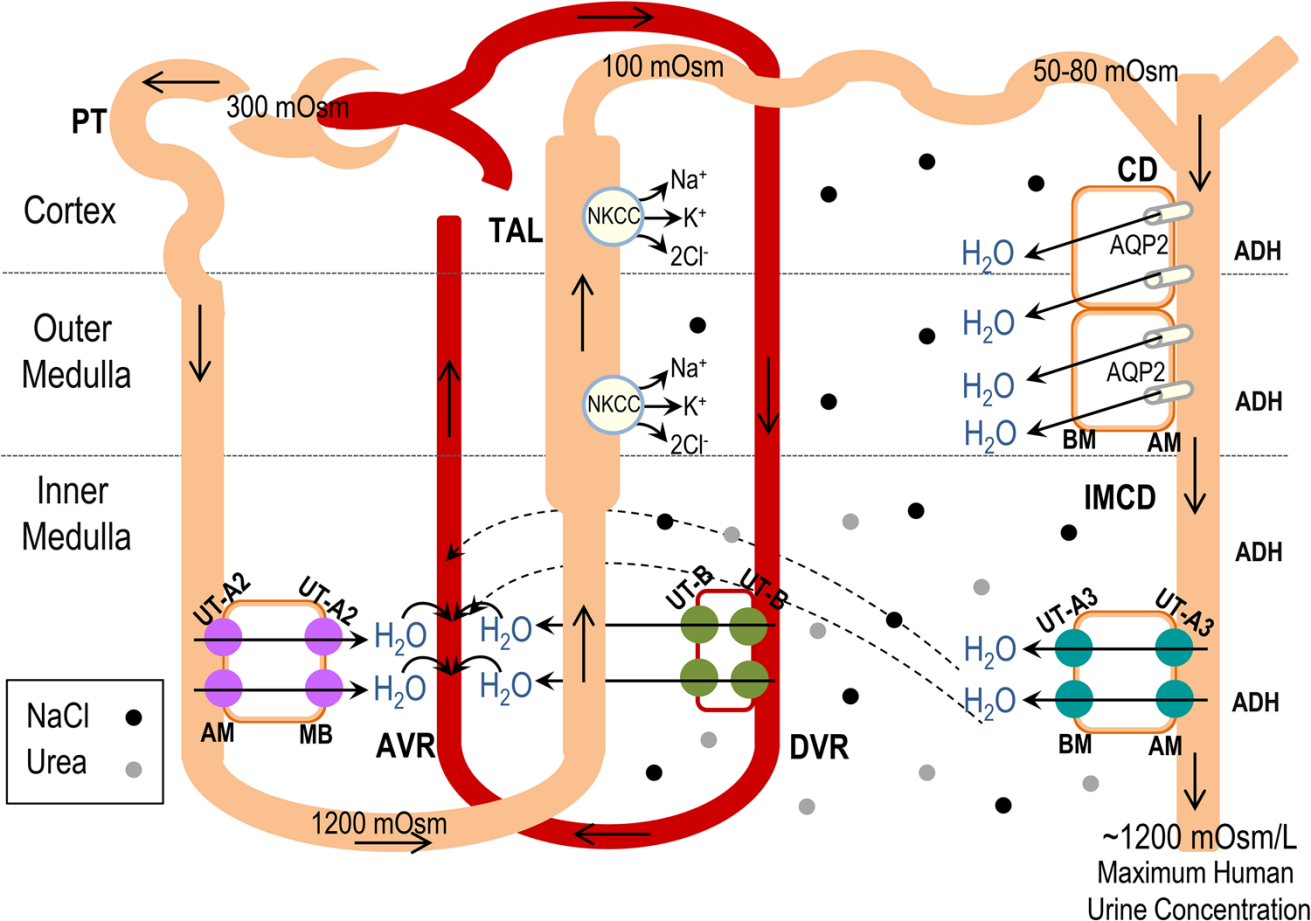
**Illustrated model of potential UT-mediated water transport contributions to the production of concentrated urine in the human renal inner medulla.** PT, proximal tubule; TDL, thin descending limb; TAL, thick ascending limb; CD, collecting duct; IMCD, inner medullary collecting duct; DVR, descending vasa recta; AVR, ascending vasa recta; AM, apical membrane; BM, basolateral membrane. Na^+^-K^+^-2Cl^−^ cotransporter (NKCC2). AQP2 is the water channel aquaporin 2, regulated by ADH. Black and grey spheres represent NaCl and urea, respectively. The solid and dashed arrows represent the movement of water.

Furthermore, based on the proposed localisation and demonstrated urea and water transport activities, we have proposed a model to illustrate how UTs-mediated water transport likely contributes to the production of concentrated urine. As shown in Fig. 6, NaCl is reabsorbed by the TAL of the Loop of Henle through the cotransporter NKCC2 (Ares et al., 2011). This provides NaCl to increase the osmolarity of the medullary interstitium and, at the same time dilutes the tubular fluid in the Loop of Henle. When the diluted tubular fluid reaches the CD system, facing the medullary interstitium, with its high levels of NaCl and urea (Giebisch et al., 2017), water is then reabsorbed from the IMCD through UT-A3, and perhaps UT-A1, along with water reabsorbed from the CD via AQP2. The high osmolality of the inner medullary interstitium is also the osmotic driving force for reabsorption of water from both TDL (via UT-A2) and DVR (via UT-B). The water is efficiently removed from the interstitium by the vasa recta and returns to the bloodstream, maintaining the medullary interstitial gradient and contributing to the conservation of water in the body. Taken together the results from the present study provide insights into the role of UTs in the urinary concentration process in humans. Future studies aimed at elucidating the substrate specificity and physiological roles of UTs would not only improve our understanding of how the kidney produce concentrated urine but could potentially lead to the development of novel UT-targeted diuretics.

In addition to water transport studies, our *Lithobates* oocyte system can also be employed in investigating urea transport. The observed mUT-B, mUT-A2 and mUT-A3-mediated water movement across the oocyte plasma membrane provides new and important insights into the renal mechanisms for regulating urine concentration.

## MATERIALS AND METHODS

### Heterologous expression in *Lithobates* oocytes

#### cRNA synthesis

The UT-A3 (AF258602), UT-A2 (AF367359) and UT-B (AF448798) were a gift from Dr Bryce MacIver, Harvard Medical School, MA, USA. DNA sequences encoding C-terminally c-Myc tagged murine UTs: mUT-B, mUT-A2 and mUT-A3 were subcloned into the P7TS expression vector. The resulting plasmids were transformed into TOP10 competent cells via heat shock, and purified with a DNA Miniprep kit (part #28104, Qiagen, Valencia, CA, USA). All of the plasmids were sequenced using the BigDye Terminator v3.1 Cycle Sequencing kit (part #4337455, Applied Biosystems, Foster City, CA, USA) and an ABI Prism 3130XL Genetic Analyzer (HITACHI, Tokyo, Japan).

All UT-encoding cDNAs were linearised with *Xba*I restriction enzyme (part #R0145S, New England Biolabs, Ipswich, MA, USA) and purified using the QIAquick PCR Purification Kit (part #27106, Qiagen). The linearised and purified DNAs were transcribed into capped RNA (cRNA) using the T7 mMachine Kit (part #AM1344, Ambion, Austin, TX, USA) and the cRNAs were purified with the RNeasy MinElute RNA Cleanup Kit (part #74204, Qiagen). The concentration and purity of all DNAs and RNAs were quantified using a Nanodrop 2000c spectrophotometer (Thermo Fisher Scientific, Waltham, MA, USA).

#### Animals

All surgical and experimental procedures involving animals were previously approved by the Committee of Animal Care and Use at the Institute of Biomedical Sciences of the University of Sao Paulo, Brazil (protocol #7971160519). Briefly, adult female *L. catesbeianus* were purchased from ‘Rã’s’ World (Sao Paulo, SP, Brazil), maintained in an aquatic tank (temperature 22°C), fed a protein-based diet (Poli-Nutri, Sao Paulo, Brazil) and exposed to a 12:12 h light:dark cycle. The frogs chosen for surgery weighed between 350–450 g.

#### Solutions

As reported previously (Kabutomori et al., 2018; Musa-Aziz et al., 2010), the osmolarity of all the solutions was adjusted to 195 mosmol/L using NaCl or water, and the pH was adjusted to 7.50 with NaOH or HCl. Standard ND96 solution contained (in mM) 96 NaCl, 2 KCl, 1 MgCl_2_, 1.8 CaCl_2_, and 5 HEPES. The 0 Ca^2+^ solution is a modified version of ND96, in which the CaCl_2_ was removed and replaced with NaCl. The OR3 media contained 6.85 g/l of Leibovitz L-15 cell culture medium (GIBCO-BRL, Gaithersburg, MD, USA), antibiotics [10,000 U/ml penicillin and 10,000 mg/ml streptomycin (GIBCO-BRL)] and 5 mM HEPES. For urea uptake experiments, the standard ND96 solution was supplemented with 5 µCi of radioactive [^14^C] urea and 1 mM of unlabelled urea (Geyer et al., 2013a). For osmotic water permeability (*P*_f_) assays, the standard ND96 solution was diluted with water to prepare a hypotonic ND96 variant (70 mosmol/L) (Geyer et al., 2013b; Kabutomori et al., 2018; Musa-Aziz et al., 2009). For inhibitory studies, 0.5 mM Phloretin (part #P7912, Sigma-Aldrich, St Louis, MO, USA) was dissolved into standard ND96 solution and used immediately (Geyer et al., 2013a). A final pH of 7.50 was verified after solution preparation.

#### Surgery

Following anesthesia by submersion into a solution of 0.2% 3-aminobenzoic acid Ethyl Ester (Tricaine) (Sigma-Aldrich) in 5 mM Hepes, pH 7.50, the frogs were placed in an ice-filled container on a cold platform. Then a 1–1.5 cm incision was made laterally to the midline of the abdomen and fragments of the ovaries for oocyte isolation were surgically removed (Kabutomori et al., 2018).

#### Isolation of *Lithobates* oocytes

Oocyte isolation was performed according to Kabutomori et al. (2018) and involved exposing the ovary fragments to collagenase type VII (0.25 mg/ml) (part #C077, Sigma-Aldrich) in the 0-Ca^2+^ solution at room temperature for 5 min. Following enzymatic digestion, strong and robust stage V-VI oocytes were separated from the less mature or dead oocytes, and mechanically defolliculated using two watchmaker's forceps. Oocytes were placed in OR3 media (part #15140-122, Gibco, Grand Island, NY, USA) and stored at 18°C until needed.

#### Microinjection of cRNAs

One day after oocyte isolation, individual oocytes were injected with either 25 nL of mUT-B, mUT-A2 or mUT-A3 cRNA (concentration 1 µg/µl), or an equivalent volume of sterile water. All injections were performed using an injection needle pulled with a Model P-97 Flaming/Brown micropipette puller (Sutter Instrument Company, Novato, CA, USA). Prior to use, the tips of the injection needles were aseptically cut to produce a tip that was approximately 2 μm in diameter (Kabutomori et al., 2018; Musa-Aziz et al., 2010). Injections were performed with mineral oil (part #M5904, Sigma-Aldrich) filled needles placed onto a Nanoliter 2000 volume microinjector (World Precision Instruments, WPI, Sarasota, FL, USA). All cRNA-injected and H_2_O-injected oocytes were stored in OR3 media, at 18°C. Routinely, the protein expression and function experiments below were performed 4 days after injection.

### Membrane expression

#### Biotinylation

UT- and H_2_O**-**injected oocytes were biotinylated using the EZ**-**Link Sulfo**-**NHS**-**Biotinylation kit (part #89881, Thermo Fisher Scientific), as previously described (Geyer et al., 2013a; Kabutomori et al., 2018). Before performing the labelling experiments, the PBS (part #28372, Thermo Fisher Scientific) and TBS (part #28379, Thermo Fisher Scientific) solutions provided with the kit were diluted to 195 mOsm/Ls, so as to match the osmolality of the oocytes. For each independent experiment, 20 UT-injected or H_2_O-injected control oocytes were transferred to a solution of PBS containing 0.24 mg/ml of EZ-link-sulfo-NHS-Biotin (part #21425 Thermo Fisher Scientific) and incubated at 4°C for 1 h. The labelling reactions were terminated by adding 250 µl of the Quenching solution provided in the kit. Next, the oocytes were washed in TBS and lysed in 200 µl of lysis buffer [TBS, 1% TX-100 and cOmplete Mini EDTA-free protease inhibitors (part #04693124001, Roche, Indianapolis, IN, USA)] by repeatedly pipetting the oocytes up and down in a P200 pipette tip. The homogenised samples were centrifuged at 3000×***g*** at 4°C for 10 min, and the supernatant was transferred to a new Eppendorf tube.

Total protein expression was assessed by mixing 20 µl of the supernatant with 2× sample buffer (1:1 ratio), and surface expression was evaluated using the eluted material collected after incubating 180 µl of the supernatant with 180 µl of NeutrAvidin (part #1859388, Thermo Fisher Scientific) in a sealed Spin X column (part #8163, Corning, Pittston, PA, USA) at room temperature with continuous mixing for 1 h. After washing the samples three times with lysis buffer the biotinylated proteins were eluted from the resin by adding 180 µl of elution buffer (1× sample buffer and 0.5 M DTT), incubating the columns on a rocker platform at room temperature for 1 h, and centrifuging the columns and collection tubes at 1000×***g*** for 1 min.

#### Immunoblots

Total and surface protein samples were first separated using 12% Tris-glycine SDS-PAGE gels and then transferred to PVDF membranes. The membranes were blocked with TBST plus 5% powdered milk (TBST-B) at room temperature for 1 h. Next, the membranes were incubated with a primary monoclonal anti-C-myc antibody (part #1849372, Invitrogen, Carlsbad, CA, USA) at 4°C, overnight. After thoroughly washing the blots with TBS, a secondary goat anti-mouse antibody (part #041806, KPL, Gaithersburg, MD, USA) was included with the blots and incubated at room temperature for 1 h. Immunoreactive bands were visualised by applying the ECL plus western blotting detection reagents (part #32132, Thermo Fisher Scientific) and the images were captured with an Amersham Imager 600 (GE Healthcare Life Sciences, Logan, Piscataway, NJ, USA).

### Physiological measurements

#### Urea uptake

Oocyte urea transport activity was measured by monitoring [^14^C] urea uptake (Geyer et al., 2013a). Briefly, groups of five oocytes (UT-injected or H_2_O-injected) were placed in 200 μl of ND96 containing 5 μCi of [C^14^] (part #NEC108V250UC, PerkinElmer, Waltham, MA, USA) urea plus 1 mM of unlabeled urea. After 10 min, the oocytes were washed in ND96 solution containing 1 mM of unlabeled urea. Each oocyte from each group (control and experimental) was lysed in 100 μl of a 5% SDS solution using a P200 pipet tip and transferred to a vial containing 5 ml of scintillation fluid for [C^14^] analysis (Geyer et al., 2013a). When investigating inhibition, oocytes were pre-incubated in ND96 plus 0.5 mM of phloretin (195 mOsm, pH 7.50) for 20 min before the [C^14^] urea solution addition.

#### Osmotic water permeability

Oocyte *P_f_* was determined using a video microscopy approach that can monitor changes in cell volume (Geyer et al., 2013b; Kabutomori et al., 2018). Groups containing six oocytes were placed in a hypotonic ND96 solution (70 mOsm/L H_2_O) and the cell swelling was monitored using a Nikon stereoscopic microscope (SMZ 745T) connected to a digital camera (Optix Cam, Roanoke, VA, USA). As a reference, a 1.3 mm in diameter metallic ball was placed close to the oocytes. Images were collected every second for 100 s. The *P_f_* (cm/s) was calculated based on the change in image density over time. For the inhibition experiments, the oocytes were pre-incubated in ND96 plus 0.5 mM of phloretin (195 mOsm, pH 7.50) for 20 min. before being osmotically challenged with the hypotonic ND96 variant solution.

### Statistics

All data are presented as the mean±standard error of the measurement (s.e.m.). Standard two-tailed Student's *t*-tests were performed when comparing the difference between two means and the level of significance was set at *P*<0.05. Student's *t*-tests with Bonferroni correction were performed when comparing more than two means and the level of significance was set at *P*<0.0125. Statistical analyses were performed using the Synergy Software version 4.0 (Synergy Software, Reading, PA, USA).
